# Nitrogen fixation by cyanobacteria stimulates production in Baltic food webs

**DOI:** 10.1007/s13280-015-0660-x

**Published:** 2015-05-28

**Authors:** Agnes M. L. Karlson, Jon Duberg, Nisha H. Motwani, Hedvig Hogfors, Isabell Klawonn, Helle Ploug, Jennie Barthel Svedén, Andrius Garbaras, Brita Sundelin, Susanna Hajdu, Ulf Larsson, Ragnar Elmgren, Elena Gorokhova

**Affiliations:** Department of Environmental Science and Analytical Chemistry, Stockholm University, 106 91 Stockholm, Sweden; Department of Ecology, Environment and Plant Sciences, Stockholm University, 106 91 Stockholm, Sweden; AquaBiota Water Research, Löjtnantsgatan 25, 115 50 Stockholm, Sweden; Department of Biology and Environmental Sciences, University of Gothenburg, Box 461, 405 30 Gothenburg, Sweden; Mass Spectrometry Laboratory, Center for Physical Sciences and Technology, Savanoriu 231, 02300 Vilnius, Lithuania

**Keywords:** Diazotrophic cyanobacteria, Food webs, Zooplankton grazing, Secondary production, Fish

## Abstract

Filamentous, nitrogen-fixing cyanobacteria form extensive summer blooms in the Baltic Sea. Their ability to fix dissolved N_2_ allows cyanobacteria to circumvent the general summer nitrogen limitation, while also generating a supply of novel bioavailable nitrogen for the food web. However, the fate of the nitrogen fixed by cyanobacteria remains unresolved, as does its importance for secondary production in the Baltic Sea. Here, we synthesize recent experimental and field studies providing strong empirical evidence that cyanobacterial nitrogen is efficiently assimilated and transferred in Baltic food webs via two major pathways: directly by grazing on fresh or decaying cyanobacteria and indirectly through the uptake by other phytoplankton and microbes of bioavailable nitrogen exuded from cyanobacterial cells. This information is an essential step toward guiding nutrient management to minimize noxious blooms without overly reducing secondary production, and ultimately most probably fish production in the Baltic Sea.

**Electronic supplementary material:**

The online version of this article (doi:10.1007/s13280-015-0660-x) contains supplementary material, which is available to authorized users.

## Introduction

### Nitrogen-fixing cyanobacteria are natural to the Baltic Sea

Cyanobacterial blooms are well known in tropical oceans and in freshwater lakes around the world. They also occur in the Baltic Sea, one of the largest areas of brackish water in the world, where large blooms of filamentous cyanobacteria appear every summer (Wasmund 1997). These cyanobacteria fix dissolved N_2_ (diazotrophy), thus circumventing a general nitrogen limitation of the primary production (Granéli et al. 1990), and have been present in the Baltic Sea for c. 7000 years (Bianchi et al. 2000). The prevailing view is that the blooms are a nuisance at best and harmful at worst. Their nitrogen fixation adds large amounts of nitrogen exacerbating eutrophication in the system (Larsson et al. 2001; Gustafsson et al. 2013). Cyanobacterial blooms are often considered to have increased in frequency and magnitude in the Baltic Sea in recent decades (e.g., Kahru and Elmgren 2014). A sequence of events has been proposed to explain this: a higher nutrient load (both N and P) leading to increases in spring phytoplankton production (Gustafsson et al. 2012) and subsequently in sedimentation of organic material, leading to more widespread bottom anoxia in the Baltic (Conley et al. 2011). When sediments turn anoxic, they release stored P (Gunnars and Blomqvist 1997), the primary limiting nutrient for N-fixing cyanobacteria (Walve and Larsson 2007). Little consideration is usually given to conditions promoting control of the bloom initiation, whether by pelagic or benthic grazers.

### Cyanobacteria–consumer linkages

The filamentous diazotrophic cyanobacteria *Nodularia spumigena* Mertens, *Aphanizomenon* sp., and *Dolichospermum* (formerly *Anabaena*) spp. are the major components of cyanobacterial blooms in the Baltic Sea (Niemi 1979; Wasmund 1997). As most cyanobacteria, they produce numerous bioactive compounds and toxins (e.g., Sivonen and Jones 1999) and these bloom-forming cyanobacteria are therefore considered potentially harmful (Karjalainen et al. 2007). *N. spumigena* is usually of major concern, because it produces the hepatotoxin nodularin (Sivonen and Jones 1999), which makes up c. 90 % of cyano-hepatotoxins in the Baltic (Kankaanpää et al. 2009) and is detectable throughout the food web (e.g., Karjalainen et al. 2007). Baltic strains of *Dolichospermum* spp. produce microcystins, likewise hepatotoxins, whereas no such toxin has been reported in Baltic strains of *Aphanizomenon* (El-Shehawy et al. 2012).

Today, nutrient management decisions are based on the assumption that production of filamentous toxin-producing cyanobacteria cannot be efficiently utilized by grazers (Elmgren and Larsson 2001) and thus do not contribute to fish production (but see Hansson et al. 2007). Evidence is, however, accumulating that these cyanobacteria are eaten by many grazers, suspension-feeders, and deposit-feeders, often with beneficial effects on their growth and reproduction. In line with this, field studies show that organic matter and bioavailable nitrogen produced by these cyanobacteria are incorporated by pelagic, littoral, and benthic food webs (e.g., Rolff 2000; Karlson et al. 2014; Lesutiene et al. 2014), challenging the view that cyanobacteria do not contribute to secondary production. Moreover, grazing on toxic cyanobacteria is species-specific and influenced by many factors (e.g., toxin concentrations and availability of other prey), with concomitant feed-back effects on toxin production (Gorokhova and Engström-Öst 2009; Engström-Öst et al. 2011). Therefore, changes in the relative abundance of grazers may alter the composition and toxicity of the cyanobacterial assemblages. Also, benthic fauna may influence the initiation and species composition of the blooms, since *Dolichospermum* spp. and to some extent also *N*. *spumigena* depend on recruitment from benthic resting stages, akinetes, which are deposited in sediments after the bloom (Suikkanen et al. 2010) and can be affected by deposit-feeders (Karlson et al. 2012).

**Fig. 1:**
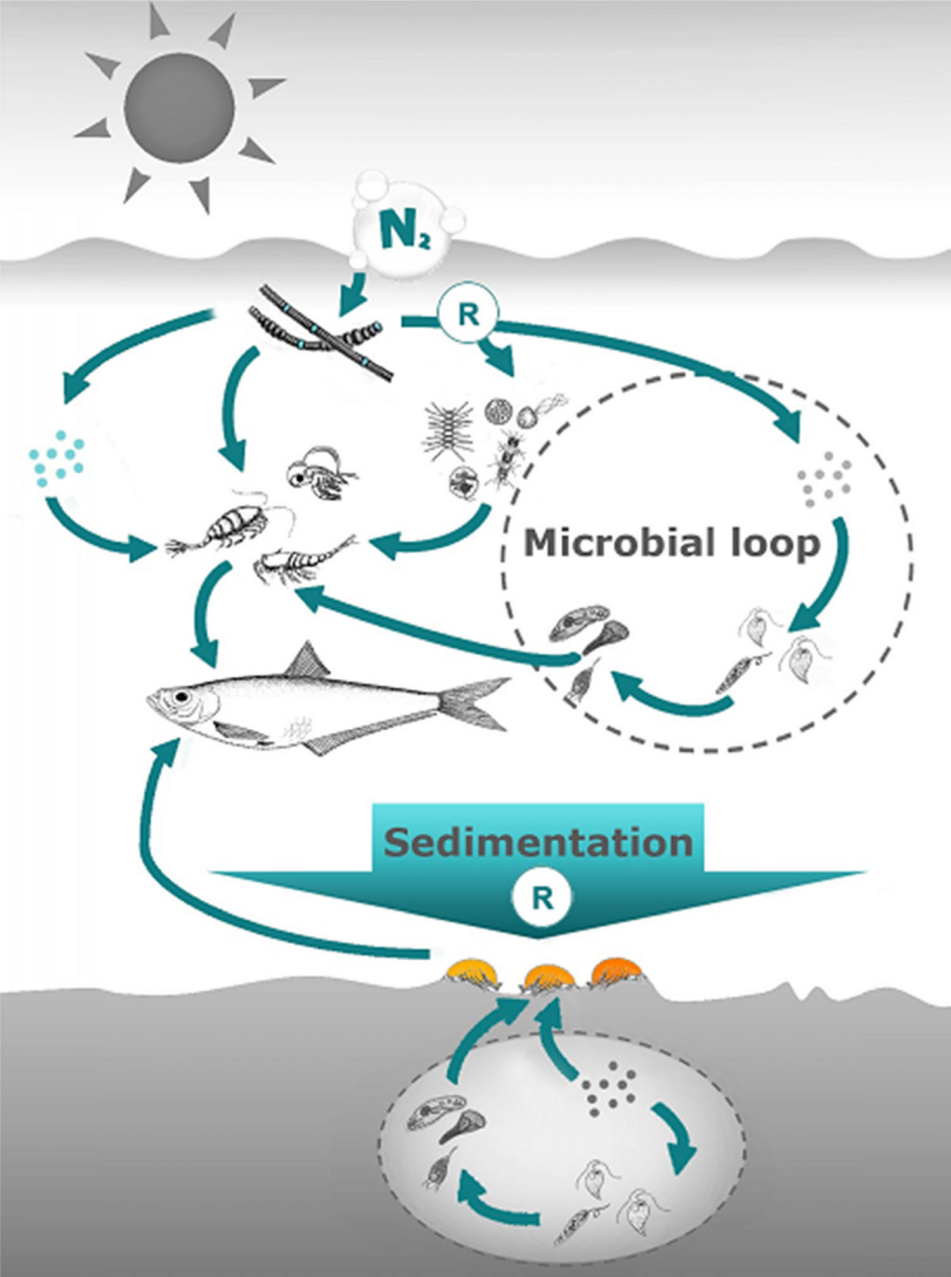
Bloom-forming diazotrophic cyanobacteria stimulating secondary production in the Baltic Sea. By fixing dissolved N_2_, these cyanobacteria are important suppliers of bioavailable nitrogen to the pelagic and benthic food webs that support fish production. The bioavailable nitrogen enters the food web through direct grazing on fresh or decaying filamentous cyanobacteria by various invertebrates and by cyanobacterial cells releasing bioavailable nitrogen (*denoted by* *R*) that is taken up by other phytoplankton and microbes, which are in turn eaten by animals in the water column and sediments

This review synthesizes recent studies addressing utilization of cyanobacterial production in pelagic and benthic food webs that collectively provide strong evidence that cyanobacterial nitrogen is efficiently assimilated and transferred in Baltic food webs (Fig. 1). To avoid overly reducing secondary production and prey availability for fish, quantification of these trophic pathways is desirable for predicting effects of nutrient reduction aiming to reduce cyanobacterial blooms.

## Biogeochemical role of cyanobacteria in the Baltic Sea

### Nitrogen fixation as a source of bioavailable N in the ecosystem

In summer, the Baltic Sea proper is characterized by a thermocline at 10–20 m depth and a permanent halocline at 60–70 m that limits water mixing. Nutrients regenerated above the thermocline are immediately available for primary producers, while those regenerated below the thermocline become available in the upper mixed layer only through wind-induced mixing and upwelling. The input of new bioavailable N through N_2_-fixation (Table S1 in Supplementary Material) is concentrated to the summer. A recent study based on multiple approaches to determine new production in the Baltic proper estimated that N_2_-fixation provides 20–45 % of the yearly average new production, while external inputs of NO_2_^−^ and NO_3_^−^ provide >50 % (Gustafsson et al. 2013), which implies a yearly N_2_-fixation of 100–200 mmol N m^−2^ year^−1^ (Table S1).

### Estimates of N transfer from diazotrophic cyanobacteria to other primary producers

Baltic Sea *Aphanizomenon* sp. and *N*. *spumigena* directly release on average 20–35 % of their fixed N as ammonium, which is then used by other primary producers, e.g., picocyanobacteria, as shown by various approaches including ^13^C and ^15^N tracers (Ohlendieck et al. 2000; Stal et al. 2003; Ploug et al. 2011). Recently, nanoscale secondary ion mass spectrometry (nanoSIMS) has made it possible to measure the N- and C-fixing activity of individual cells in mixed populations of phytoplankton and microbes. While it has been suggested that Baltic Sea picocyanobacteria may fix N_2_ (Wasmund et al. 2001), nanoSIMS-based analysis demonstrated that Baltic picocyanobacteria do not fix N, but use NH_4_^+^ released from filamentous N_2_-fixing cyanobacteria. In control incubations, cells <5 µm showed high ^13^C-assimilation, but no ^15^N_2_-fixation. However, when heterocyst-bearing filamentous cyanobacteria were present, the^15^N label was rapidly transferred to picocyanobacteria, diatoms, and zooplankton (Adam et al., unpublished). Hence, these N-fixing cyanobacteria supply bioavailable N to the rest of the plankton community (Fig. S1).

### Sedimentation of cyanobacteria and fixed nitrogen

Sediment-trap measurements indicate that little of the cyanobacteria blooms reach the bottom (Bianchi et al. 2002). The gas vacuoles that make filamentous cyanobacteria buoyant supposedly lead to decaying filaments being efficiently remineralized by microbes in the water column (Sellner 1997). However, comparison of different methods for measuring vertical fluxes of particular organic carbon and nitrogen has shown that the sinking flux of organic matter to the sediments in the Baltic Proper has likely been underestimated (Gustafsson et al. 2013). Based on this, the PON sedimentation in May–September, representing most of the spring bloom and the whole summer bloom, was calculated to be 240 mmol N m^−2^ year^−1^ (J. Gelting, pers. comm). Locally, in shallow, coastal areas where winds concentrate the blooms, an even larger sedimentation is likely.

Simultaneous measurements of sinking rate and respiration on *N*. *spumigena* aggregates in the Baltic Sea show that sedimentation during decay is rapid, increasing from 4 to 44 m day^−1^ within 12 h (Ploug 2008). A high settling flux of Baltic *Aphanizomenon* sp. has occasionally been recorded also with sediment traps (Tallberg and Heiskanen 1998). Other evidences of substantial input of N-fixing cyanobacteria to Baltic sediments include the characteristic cyanobacterial isotope signatures (Bianchi et al. 2000), cyanotoxins (Kankaanpää et al. 2009), and cyanopigments (Bianchi et al. 2000), as well as akinetes (Suikkanen et al. 2010) in sediments. Pigment analysis suggests that also unicellular cyanobacteria sink out (Bianchi et al. 2002). Thus, sedimentation of cyanobacteria and detritus derived from them supplies organic matter to benthic communities.

## Trophic transfer of diazotrophic nitrogen to pelagic and benthic consumers

### Tracking transfer of cyanobacterial nitrogen in food webs

The large variety of transfer pathways and turnover rates in food webs makes it hard to quantify the flow of bioavailable N from primary producers to top consumers. However, use of stable isotope analysis (SIA), cyanotoxins, pigments, fatty acids (FAs), and molecular diet analysis has convincingly shown that many pelagic and benthic animals consume and assimilate diazotrophic nitrogen. In addition to the bulk SIA, compound-specific SIA of amino acids has been used to evaluate the transfer of diazotrophic N in food webs because it provides an internal reference to the *δ*^15^N of the primary producers, a particularly useful property when the baseline varies due to temporal variation in N-fixation (Loick-Wilde et al. 2012).

**Fig. 2:**
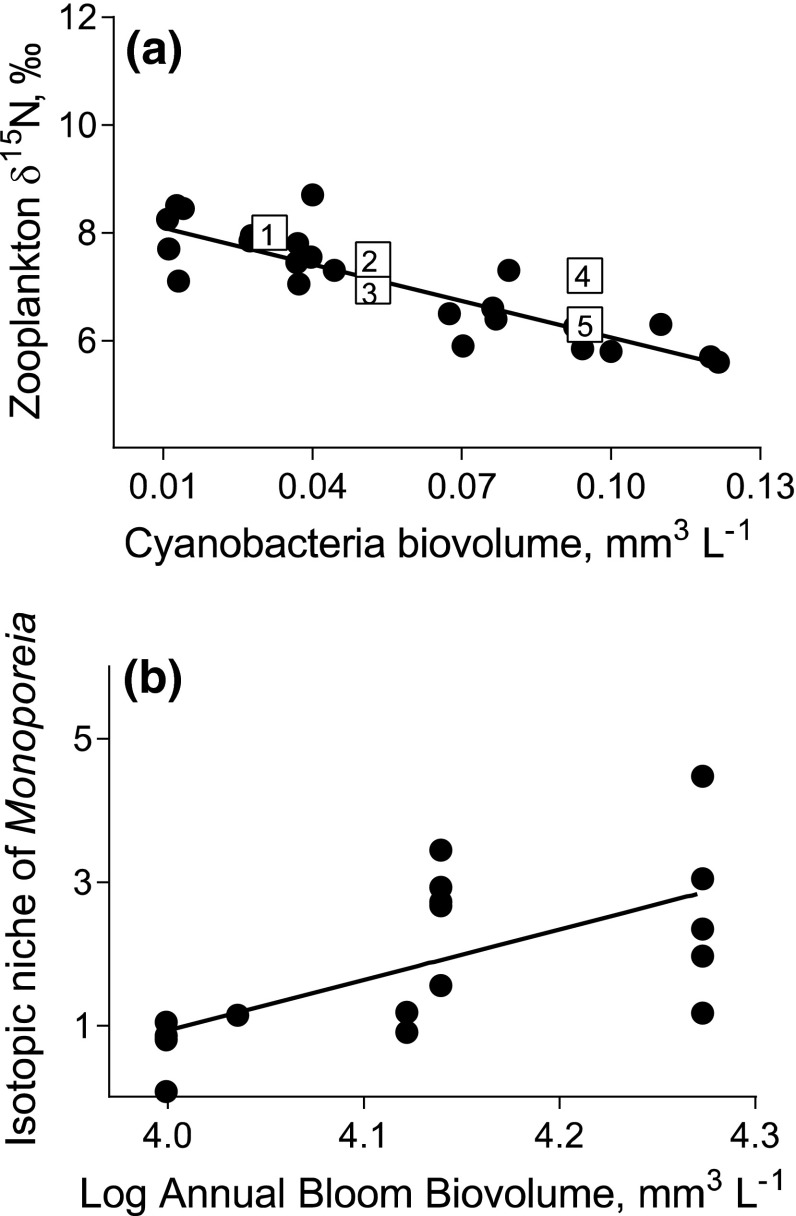
*δ* ^15^N and isotopic niche in pelagic and benthic consumers in the northern Baltic proper as a function of cyanobacterial biomass. **a** *δ* ^15^N in zooplankton (copepods and cladocerans, *filled circles*) decreases significantly with increasing mean cyanobacteria biovolume (mm^3^ l^−1^) indicating uptake of diazotrophic nitrogen by zooplankton. *Line* indicates the trend. Data for June–August, 1976–2010, Askö area, station B1; see Appendix S1 for details. Published data on *δ* ^15^N in crustacean zooplankton in summer (*open squares*) support the trend; *numbers inside the* *squares* indicate the study (*1* Hansson et al. 1997;* 2* Rolff and Elmgren 2000;* 3* Rolff 2000;* 4* Holliland et al. 2012; and* 5* Hansen et al. 2012). **b** Diet diversity measured as isotopic niche size in the deposit-feeding amphipod *Monoporeia affinis* in October increases significantly with annual bloom biovolume calculated as the area under the curve for plots of cyanobacteria biovolume over time (May to September, 2000–2011, Himmerfjärden Bay, see Appendix S2 for details). *Line* indicates the trend. Larger values of isotopic niche estimated as convex hull area suggest larger trophic diversity and greater dietary breadth. **a** Spearman *r* = −0.85, *p* < 0.001; **b** Spearman *r* = −0.79, *p* < 0.001

**Fig. 3:**
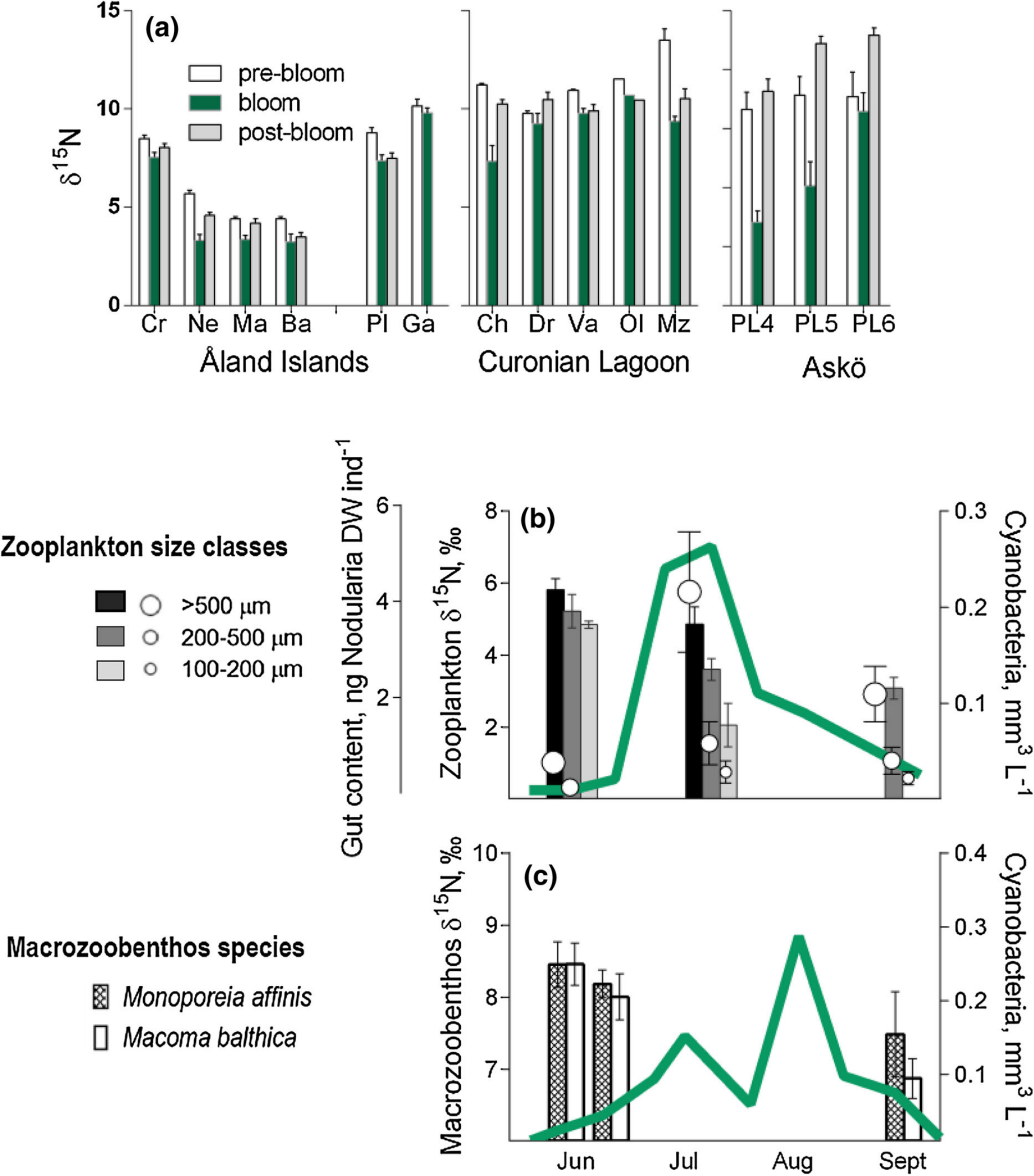
Uptake of cyanobacteria-fixed nitrogen by pelagic and benthic consumers inferred from seasonal changes in their *δ* ^15^N values and stomach content analysis in relation to the cyanobacterial bloom in different areas of the Baltic Sea. **a** *δ* ^15^N (mean ± SD) in invertebrates and fish from three coastal areas: Åland Islands (Nordström et al. 2009), Curonian Lagoon (Lesutiene et al. 2014), and Askö area (Rolff 2000), in relation to the cyanobacteria bloom. Invertebrate species and groups: *Cr* *Crangon crangon*; *Ne* *Nereis diversicolor*; *Ma* *Macoma balthica*; *Ba* *Bathyporeia pilosa*; *Ch* Chironomidae; *Dr* *Dreissena polymorpha*; *Va* Valvata; *Ol* Oligochaeta; *Mz* mesozooplankton; *PL*4 zooplankton, size 100–200 µm (nauplii, rotifers, ciliates); *PL*5 zooplankton, size 200–500 µm (copepodites, cladocerans); and *PL*6 zooplankton, size > 500 µm (adult copepods). Fish: *Pl* *Platichthys flesus* and *Ga* *Gasterosteus aculeatus*; **b** *δ* ^15^N values of zooplankton (*bars*, primary *Y*-axis on the left side; mean ± SD, *n* > 3) and grazing on cyanobacteria derived from qPCR-based estimates of *N. spumigena* abundance in zooplankton stomachs (*circles*; secondary *Y*-axis on the left side; mean ± SD, *n* > 4) sampled throughout cyanobacteria bloom (*green line*) in the open sea (Landsort Deep, station BY31; year 2011; Motwani 2015; see Appendix S3). **c** *δ* ^15^N values in deposit-feeders (mean ± SD, *n* > 10) sampled before and after a cyanobacteria bloom (*green line*) in a coastal area (Askö, station B1; year 2010; modified from Karlson et al. (2014)

Studies tracing source and fate of N in aquatic food webs rely largely on the use of ^15^N. Diazotrophic cyanobacteria that use N_2_ gas to satisfy their nitrogen requirements have *δ*^15^N values between −1 and −2 % (e.g., Rolff 2000). This isotopic signature can be used to track the flow of diazotrophic N through the food web (Figs. 2, 3). Early studies tracked the flow of cyanobacteria-derived N through pelagic primary and secondary trophic levels in the Baltic Sea using the depleted ^15^N-signal (Meyer-Harms et al. 1999; Rolff 2000). Later studies show that many invertebrates and fish become ^15^N depleted during cyanobacterial blooms, indicating transfer of diazotrophic N in the food web (Fig. 3a). This effect of cyanobacteria on *δ*^15^N values in benthic and pelagic consumers is a combined result of direct consumption of cyanobacteria, secondary consumption of fixed N via microbial food webs, and direct consumption of non-diazotrophic phytoplankton that have taken up N exuded by diazotrophs. Therefore, the observed correlation between *δ*^15^N in a primary consumer and abundance of filamentous cyanobacteria (Fig. 2a) does not prove direct feeding, but may also result from secondary transfer via multiple trophic pathways.

### Evidence of direct grazing from molecular diet analysis

Gut content provides direct evidence of trophic linkage. For relatively large consumers, such as fish and mysids, gut content analysis is the classic method for diet analysis. Recently, molecular diet analysis has made it feasible to identify and quantify Baltic cyanobacteria in the gut of consumers as small as nauplii and rotifers (*N. spumigena*: Gorokhova 2009; Engström-Öst et al. 2011; *Synechococcus*: Motwani and Gorokhova 2013), providing in situ data on direct cyanobacteria grazing by zooplankton. This approach uses the polymerase chain reaction (PCR) and species- or group-specific primers and DNA sequencing to identify prey DNA in the gut of the consumer. Using this method (Fig. S2), *N*. *spumigena* was found to be a common food for mysids (c. 58 % of population; Gorokhova 2009) and copepods (nearly 100 %; Gorokhova and Engström-Öst 2009). Moreover, mysids digest cyanobacteria, as indicated by lower yield of cyanobacterial DNA in their feces than in the stomach bolus (Gorokhova 2009). When bioavailable with SIA, a decrease in *δ*^15^N signal was observed in planktonic grazers when cyanobacteria quantity in their stomachs increases (Fig. 3b), implicating direct grazing as an important pathway. Grazing on cyanobacteria is not unique to the Baltic Sea; qPCR analysis has shown that freshwater zooplankton eat toxic cyanobacteria both in bloom (Sotton et al. 2014) and non-bloom (winter) conditions (Oberholster et al. 2006).

A qPCR assay is also available for *Synechococcus*, a large picocyanobacterial clade contributing up to half the summer phytoplankton biomass in the offshore Baltic Sea (Hajdu et al. 2007). With small size and high growth rate, picocyanobacteria are most likely to benefit from diazotroph exudates supplying N (Ploug et al. 2011). All main Baltic zooplankton groups feed on *Synechococcus* (8–10 % of the gut content in copepods and 15–30 % in cladocerans, rotifers, and nauplii; Motwani and Gorokhova 2013). This direct metazooplankton grazing on non-diazotrophic picoautotrophs, fueled by diazotroph exudates, is thus potentially an important source of N for zooplankton in summer (Fig. 1).

### Fatty acids and toxins

Cyanobacteria-derived material can also be distinguished by the presence and ratios of particular FAs (Ahlgren et al. 1992), and Baltic copepods assimilate cyanobacterial FAs (Peters et al. 2006). Finally, cyanotoxins in consumer tissues indicate feeding on cyanobacteria (Karjalainen et al. 2006; Sipiä et al. 2006; Mazur-Marzec et al. 2007). However, such linkages are uncertain, since we do not know how the toxin reached the consumer (see review in Karjalainen et al. 2007), and because animals (and their microbes) vary in their capacity to metabolize toxins (Karjalainen et al. 2006).

Combined approaches are required to estimate the relative importance of various pathways of N incorporation in food webs. For example, by combining analysis of cyanobacteria-specific pigments and *δ*^15^N in copepods, Meyer-Harms et al. (1999) showed that copepods eat filamentous cyanobacteria and assimilate fixed N. Combining SIA with PCR-, FA-, or pigment-based methods for prey identification can facilitate the use of mixing models for diet analysis by justifying inclusion of specific prey in models that estimate diet composition (Gorokhova and Lehtiniemi 2007).

## Translating cyanobacteria-driven primary production into secondary production

### Feeding on cyanobacteria can increase reproductive output and somatic growth in consumers

Like other filamentous toxin-producing cyanobacteria, Baltic cyanobacteria are generally considered to be inadequate as food for grazers due to their (i) toxins and bioactive compounds that hamper digestion, (ii) poor manageability, and (iii) low nutritional value due to low content of essential components like polyunsaturated FAs and sterols (e.g., Gulati and DeMott 1997). However, cyanobacteria are a rich source of N and P (Walve and Larsson 2007) and essential amino acids (Ahlgren et al. 1992; Loick-Wilde et al. 2012) as well as some vitamins (Prasanna et al. 2010) and antioxidants (Pandey and Pandey 2009). Also, cyanobacterial filaments host a rich community of associated microorganisms (Hoppe 1981; Ploug et al. 2011) that should increase their nutritional value.

Although Baltic zooplankton have been reported to avoid ingesting filamentous cyanobacteria, there are also reports that they feed on cyanobacteria, in the laboratory, in mesocosms, and in the field (reviewed in Hogfors et al. 2014; summarized in Fig. S2). In situ, zooplankton prefer other phytoplankton, but feed opportunistically on cyanobacteria (Meyer-Harms et al. 1999).

Short-term laboratory studies reporting negative effects of toxic *N*. *spumigena* on zooplankton survival and reproduction have used cyanobacteria cultures, often as the single food source (e.g., Engström et al. 2000, 2001). However, a meta-analysis of laboratory experiments on the effects of cyanobacteria on zooplankton population growth across genera and species showed that 73 % of species maintained positive growth when fed a diet containing cyanobacteria, even though cyanobacteria were a poorer food than green algae and/or flagellates alone for half of the species tested (Tillmanns et al. 2008). Moreover, adding other algal species of sufficient nutritional quality to a mono-diet of cyanobacteria can reduce the negative effects (Reinikainen et al. 1994).

**Fig. 4:**
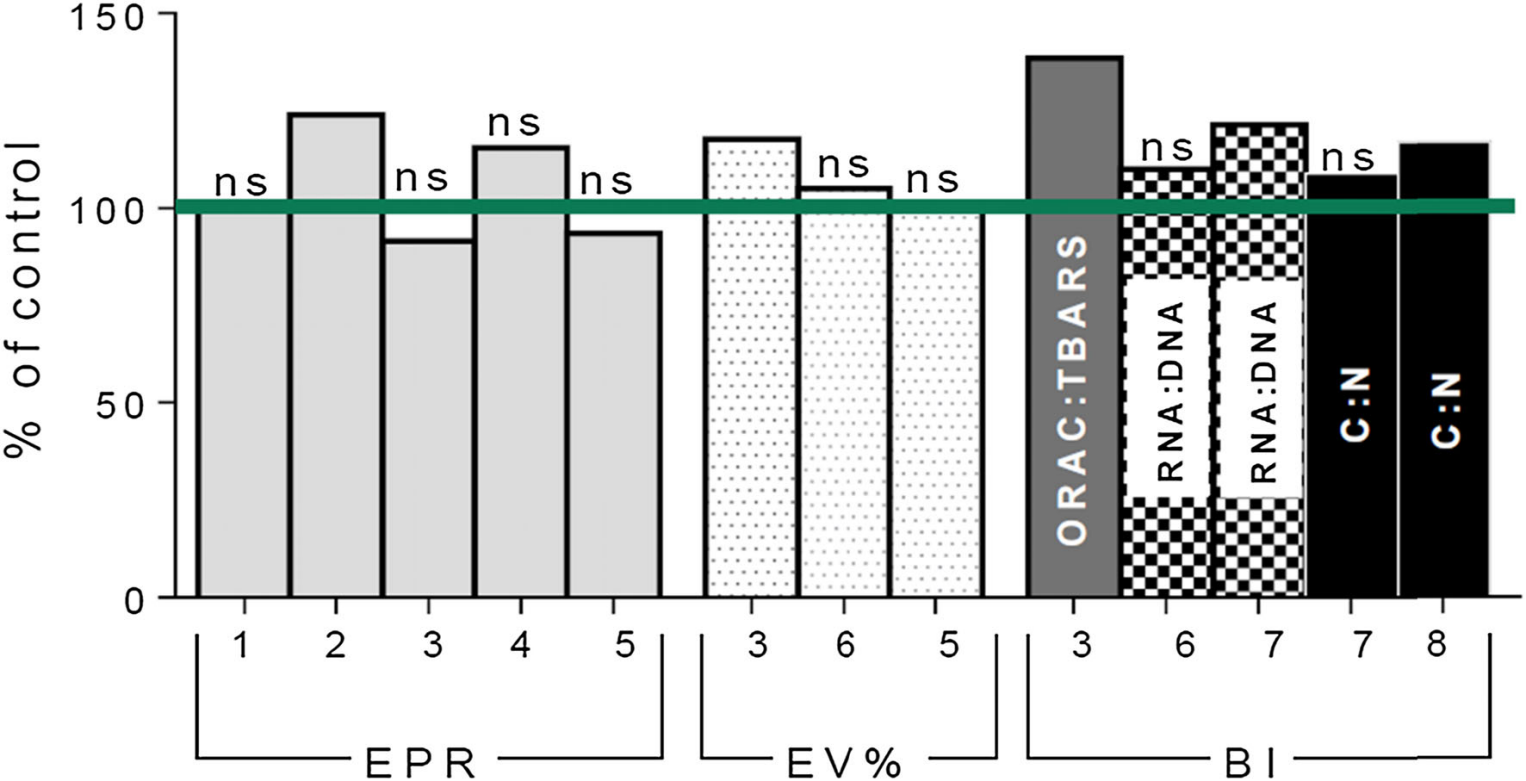
Effects of cyanobacteria on fitness-related traits (*EPR* egg production rate and *EV*% egg viability) and growth-related biochemical indices (*BI*) in Baltic copepods (*Acartia bifilosa*, *A*. *tonsa*, and *Eurytemora affinis;* studies* 1*–*4*, and* 6*) and amphipods (*Monoporeia affinis*; studies* 5* and* 7*–*8*). Only studies that used feeding conditions with cyanobacteria concentrations approximating those during summer bloom in the Baltic Sea and animals pre-exposed to the bloom are presented. Effects are expressed as % deviation from the control (non-cyanobacterial diet, *green line*); non-significant effects (*p* > 0.05) are marked *ns*. *Numbers on the* *X*-*axis* indicate study reporting the effect;* 1* Schmidt and Jonasdóttir (1997),* 2* Koski et al. (2002),* 3* Vehmaa et al. (2013),* 4* Schmidt et al. (2002),* 5* Wiklund et al. (2008),* 6* Hogfors et al. (2011),* 7* Karlson (unpubl.) (see Appendix S4), and* 8* Karlson et al. (2014). The biochemical indices of growth status: *ORAC*:*TBARS* ratio [*ORAC* total antioxidative status measured as oxygen radical absorbance capacity and *TBARS* lipid peroxidation measured as production of thiobarbituric acid reactive substances; (Vehmaa et al. 2013)] is a proxy for oxidative status, *RNA*:*DNA* ratio is a proxy for protein synthetic capacity, and the C:N ratio reflects lipid storage in amphipods

In line with this, studies simulating ambient feeding conditions during a cyanobacterial bloom reported that Baltic copepods can ingest cyanobacteria without negative effects on survival, egg production, or hatching success (Fig. 4). Moreover, fitness-related parameters responded differently to cyanobacteria addition. For example, addition of *N*. *spumigena* to a mono-diet of green alga reduced egg production, but improved oxidative balance, egg viability, and early naupliar development in *Acartia bifilosa* (Vehmaa et al. 2013). By contrast, increasing concentrations of *N. spumigena* in the copepod diet had no effect on egg hatching success, although there was a negative relationship between copepod gross growth efficiency and accumulated nodularin (Kozlowsky-Suzuki et al. 2003). The overall conclusion of both studies was that *N*. *spumigena* is not harmful to copepods if an alternative food is also available. Since N-sufficient diets were used in the experiments, the extra supply of N cannot explain these effects; instead, they may be related to complementary nutrients or microelements, present in *N*. *spumigena* but missing from the alternative food (Hogfors et al. 2014). In bloom conditions, the positive effects on naupliar development might be enhanced by picoplankton, stimulated by exudates, and efficiently used by nauplii, especially when high-quality phytoplankton are scarce (Motwani and Gorokhova 2013). Regardless of the mechanisms, the positive effect on egg and naupliar development should improve copepod recruitment and, ultimately, population growth, although detecting these effects at high fish predation is challenging.

In experiments with benthic deposit-feeders, cyanobacteria are generally growth neutral, whereas sediment supplied with diatoms supports rapid growth (Karlson et al. 2008; Nascimento et al. 2009; Karlson and Mozuraitis 2011). Deposit-feeders fed *Aphanizomenon* and *N. spumigena* incorporated large quantities of cyanobacterial C and N, without increased mortality (Karlson et al. 2008; Nascimento et al. 2008; Karlson and Mozuraitis 2011). Short-term experiments with relatively slow-growing animals may underestimate the value of cyanobacteria as food over longer times. In field and experimental studies mimicking in situ conditions, bivalves and amphipods fed cyanobacteria perform better than when starved (Basen et al. 2012; Gergs et al. 2014; Fig. 4), and the nutritional quality of cyanobacterial matter improves with time through toxin breakdown by animals (Svensen et al. 2005) or as a result of trophic upgrading by bacteria (Mazur-Marzec et al. 2009) or metazoans (Basen et al. 2013). Microbes contributing to such trophic upgrading should also transfer diazotrophic N to deposit-feeders (Fig. 1).

To study the effect of cyanobacterial food on a benthic deposit-feeder community, Karlson et al. (2014) used an isotope niche approach based on SIA (Layman et al. 2007). Settling material from the cyanobacterial bloom expanded the isotopic niches in deposit-feeders reflecting their trophic niche increase (Fig. 2b), with concomitant positive effects on body condition in some species, and decreased food competition (Karlson et al. 2014; Fig. 4). Moreover, species composition of the benthic community and cyanobacterial assemblages influenced the trophic transfer (Nascimento et al. 2008; Karlson et al. 2010), often with higher uptake for *Aphanizomenon* than for *N. spumigena* (amphipods: Karlson et al. 2008; mysids: Engström et al. 2001; cladocerans: Wannicke et al. 2013).

### Consequences for food web efficiency and productivity in the Baltic Sea

**Fig. 5:**
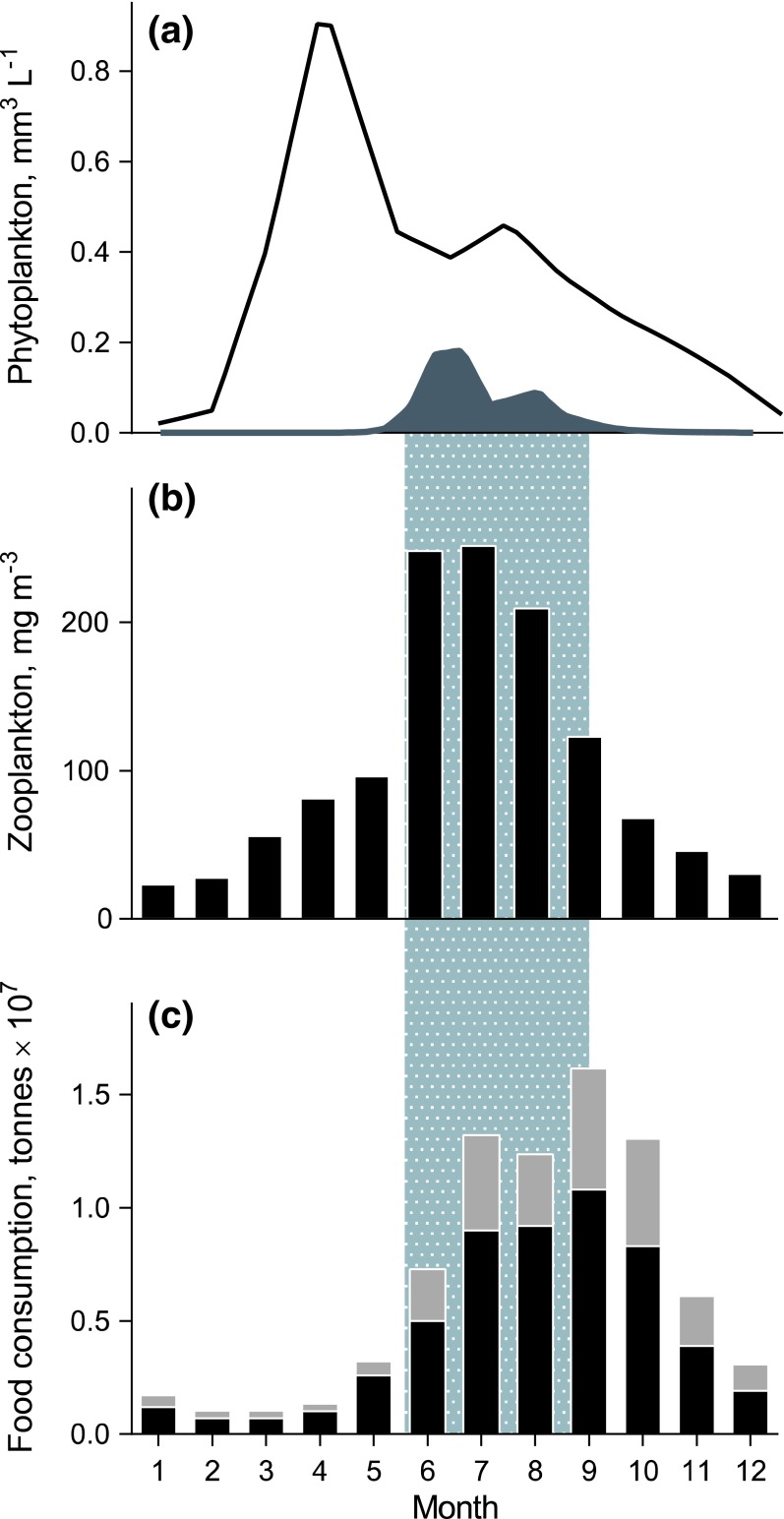
Seasonal development of **a** phytoplankton biomass, including cyanobacteria, **b** zooplankton biomass, and **c** estimated food consumption by zooplanktivorous fish in the Baltic Sea. In June–September, cyanobacteria contribute substantially to the phytoplankton communities, which coincides with the highest zooplankton stocks, and the highest food consumption by fish. The phytoplankton and zooplankton data are long-term means (1992–2011) for the Askö area (station B1), and fish estimated food consumption data are from Arrhenius and Hansson (1993). See Appendix S1 for details on plankton sampling and analysis. **a** *Black line* total phytoplankton, *grey shading* cyanobacteria; **c** *grey shading* sprat, *black shading* herring

Application of various tracers demonstrates that biologically fixed cyanobacterial N is incorporated by multiple trophic levels in the Baltic, the Gulf of Mexico (Dorado et al. 2012), and estuaries in Australia (Woodland et al. 2013). Baltic cyanobacteria rapidly build up biomass in early summer, when the pelagic food web becomes N-limited and when zooplankton production is of particular importance for the newly hatched larvae of many fish species (Hansson et al. 2007; Fig. 5). According to our current understanding of Baltic trophodynamics, supported by estimates of diazotrophy in pelagic and littoral food webs (Fig. 3), diazotrophic N enters food webs already at bloom initiation and is transferred via multiple pathways to zooplankton and benthos and, ultimately, to fish. Moreover, deposit-feeders below the photic zone are critically dependent on settling phytoplankton for growth and exhaust much of the spring bloom input within a few months (Uitto and Sarvala 1991). While the cyanobacteria are nutritionally inferior to the diatom-rich spring bloom, they are still valuable as a supplementary food during summer, and sedimentation of cyanobacteria might provide a crucial food input to benthos before winter starvation (Karlson et al. 2008; Nascimento et al. 2008). Thus, diazotrophic N supports production of zooplankton and benthos in this N-limited system during summer.

Estimating the quantitative contribution of fixed N to the secondary production is, however, difficult and requires some as yet untested assumptions (e.g., of number of trophic transfers, isotopic fractionation, and equilibrium time). Estimates vary from 5–10 % for deposit-feeders in coastal areas of the northern Baltic Proper (Karlson et al. 2010) to 50–80 % in planktonic and benthic invertebrates in the shallow Curonian Lagoon (Lesutiene et al. 2014). Mesocosm-based measurements suggest that diazotrophic bloom contributes 23–45 % to mesozooplankton biomass (Sommer et al. 2006). Moreover, using ^15^N-labeled cyanobacteria, Wannicke et al. (2013) estimated that diazotrophic N contributed 27 % to mesozooplankton production, one-third from direct grazing on filamentous cyanobacteria and the rest via the microbial loop (Fig. 1). The exact pathway can make a large difference when calculating cyanobacteria contribution to secondary production, because each added trophic step lowers the trophic efficiency. Since trophic efficiency is, therefore, an order of magnitude higher in a phytoplankton-based food web than in one based on bacteria (Berglund et al. 2007), the isotopic signal mediated by phytoplankton will be stronger and less affected by metabolic losses than in the microbial loop with its multiple trophic transfers.

The plausible positive effects of diazotrophic N on secondary production in the Baltic Sea, including the temporal coupling between nitrogen fixation, zooplankton dynamics, and the peak in zooplankton consumption by clupeids, suggest that this N input is important for fish production (Hansson et al. 2007). Since herring and other pelagic fish are food limited at this time of the year (Arrhenius and Hansson 1993), any increase in prey availability will ultimately translate into increased fish production, as also indicated by *δ*^15^N signal in some fish species following the bloom (Nordström et al. 2009; Lesutiene et al. 2014). In freshwater, application of FA markers has provided compelling evidence that toxic cyanobacterial blooms provide juvenile perch with components of high nutritional value (Perga et al. 2013). The authors concluded that the cyanobacterial bloom could be regarded as a significant dietary bonus for juvenile fish at a critical time of the year. Although the duration of this effect might be relatively short, it occurs at a crucial time for larval growth and hence fish recruitment.

While cyanotoxins are potentially harmful (particularly to vertebrates) and cyanobacterial blooms may decrease fish survival and fitness, either directly (i.e., acute toxicity) or indirectly (e.g., toxin transfer via prey, bloom-induced turbidity reducing prey capture; Karjalainen et al. 2007; Persson et al. 2011), more field studies are needed to understand the net effect of cyanobacterial blooms on fish in the Baltic Sea. Specifically, the likely benefits of diazotrophic N supporting nutrition and survival of larvae and juveniles need to be evaluated in relation to potential toxicity-induced losses in fish production. Similarly, if the benefits of ingesting cyanobacteria are greater than the costs associated with detoxification, selection will favour grazers with greater capacity to consume cyanobacteria. As cyanobacteria blooms are often predicted to increase in response to climate change (Paerl and Fulton 2006), we may expect an increasing evolutionary pressure on all Baltic food web components to cope with cyanobacteria in the system, a higher degree of co-existence for consumers and cyanobacteria and a more efficient channeling of cyanobacteria biomass to the secondary production. This is an exciting field requiring joint efforts of ecologists and evolutionary biologists.

## Implications for adaptive management of Baltic Sea ecosystems

### Knowledge gaps in our understanding of cyanobacteria importance for Baltic productivity

The main challenge for management of cyanobacteria blooms in the Baltic Sea is to establish ecological threshold levels for the occurrence of the dominant cyanobacteria, where the beneficial effects on secondary and tertiary productivity override detrimental effects on growth and reproduction of pelagic and benthic animals as well as negative effects on tourism and recreation. Quantifying the contribution of cyanobacterial blooms to production of zooplankton, benthos, and fish is critical for determining these thresholds. Future studies also need to consider other ecologically important factors, such as climate-related changes in physics, chemistry, and phytoplankton (e.g., Vehmaa et al. 2013; Kahru and Elmgren 2014), combined effects of cyanobacterial toxins and environmental contaminants (e.g., Turja et al. 2014), and changes in community structure (e.g., biological invasions) that may alter the efficiency of cyanobacteria incorporation in the food webs (Karlson et al. 2014). Integrating these factors would make a major contribution to understanding and managing cyanobacteria and ecosystem productivity in the Baltic Sea.

### Managing eutrophication without reducing secondary and fish production

To decrease eutrophication and cyanobacteria blooms, large efforts are being made to reduce P and N inputs into the Baltic Sea (Gustafsson et al. 2012). They have already resulted in nutrient declines in some local areas of the Baltic Sea (Elmgren and Larsson 2001), and bioavailable winter concentrations of N have also decreased somewhat in the open Baltic Proper, albeit not necessarily because of the decrease in loads. However, no systematic analysis of the consequences of these reductions and the changed nutrient ratios for secondary productivity in the coastal and open-sea food webs has been undertaken so far. While the spring bloom is N-limited, the cyanobacterial summer bloom appears to be P-limited (Granéli et al. 1990). Therefore, the effects of nutrient management can differ depending on the relative extent of N or P reduction. Reduced N loads are more likely to limit the organic load to sediments by reducing the spring bloom (Gustafsson et al. 2012) and, hence, the bottom hypoxia, but also benthic production. Reduced P load would decrease the summer production of cyanobacteria and probably also the other phytoplankton relying on the supply of diazotrophic N and, consequently, secondary production during summer, leading to decreasing fish production (Hansson et al. 2007). To understand and predict food web responses to our current and possible future management actions toward eutrophication and fishery (such as bottom water re-oxygenation, chemical P-precipitation, removal of zooplanktivorous fish, and other ecosystem-scale manipulations), we must recognize that cyanobacteria are one of many natural components of this ecosystem. They need to be understood in relation to other primary and secondary producers, including fish. Models are valuable tools for testing and improving our understanding at this level of ecosystem function (e.g., Savchuk and Wulff 1999; Hansson et al. 2007; Niiranen et al. 2013), and there is a great need for measurements and observations that help improve such models. It will be important to couple the ongoing efforts to manage nutrients and fishing in the Baltic Sea to resulting changes in fish feeding conditions, if we are to understand implications of our actions for the productivity of pelagic and benthic communities that governs fish production.

## Electronic supplementary material

Supplementary material (PDF 342 kb)
