# Supplementary material for: Nitrogen fixation by cyanobacteria stimulates production in Baltic food webs
Source: Ambio. 2015 May 28;44(Suppl 3):413–26. doi: 10.1007/s13280-015-0660-x (PMC4447702; doi:10.1007/s13280-015-0660-x)
Supplement: Supplementary file 1 — Supplementary material (PDF 342 kb) [file 13280_2015_660_MOESM1_ESM.pdf]

AMBIO
Electronic Supplementary Material

This supplementary material has not been copy edited by the publisher or the editorial office.

Title: **Nitrogen fixation by cyanobacteria stimulates production in Baltic food-webs**

Authors: Agnes ML Karlson, Jon Duberg, Nisha H Motwani, Hedvig Hogfors, Isabelle Klawonn, Helle Ploug, Jennie Svedén, Andrius Garbaras, Brita Sundelin, Susanna Hajdu, Ulf Larsson, Ragnar Elmgren, Elena Gorokhova

Table S1 Baltic Proper N_2-fixation rates estimated with different approaches: Nutrient budgets[a], Acetylene Reduction Assay[b], Stable isotope tracers[c].

N_2-fixation rate (nmol N L^{-1} h^{-1})	N_2-fixation rate (mmol N m^{-2} d^{-1})	Yearly N_2-fixation rate (mmol N m^{-2} y^{-1})	Year	Reference
0.41-5.41[a]	0.21 – 2.6	14.3-214	1990-1997	Rahm et al. 2000
	0.39-0.71[b]		1993	Stal and Walsby 1998
0.2-5.59[c]			1995-1996	Ohlendieck et al. 2000
0.4-11.96[c]	0.43-0.91	140-158	1998-1999	Ohlendick et al. 2007
5.07[c]	1.7±0.6 / 7.1± 4.4	101-263	1997	Wasmund et al. 2001
<7[b]			1999	Stal et al. 2003
0.006-2.98[b,c]	0.14-1.29	22-51	1999-2000	Degerholm et al. 2008
	2.3 to 5.9	60-140[a]	1994-1998	Larsson et al. 2001
		114-279[a]	1998-2000	Gustafsson et al. 2013
		134-182[a]	2001	Wasmund et al. 2005
	0.15-2.32	138[c]	2001	Wasmund et al. 2005
		92[a]	2002	Rolff et al. 2007
0.05-2.77	0.85-1.48	70-95[c]	2013	This study

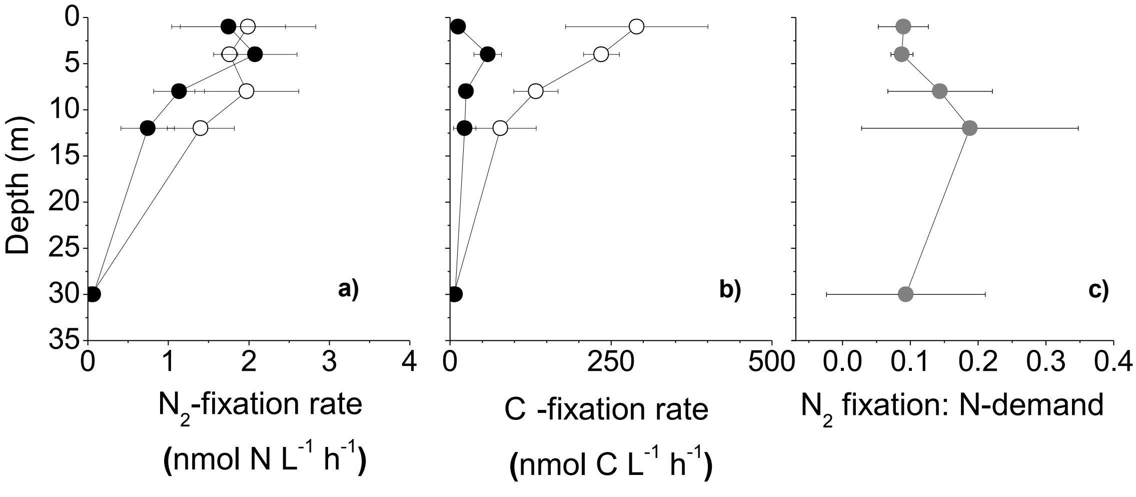

Fig. S1 Depth distributions of N_2- and C-fixation, and the ratio of N_2 fixation to N-demand during summer 2013 at the Landsort Deep, the Baltic Proper. Simultaneous measurements of C- and N_2-fixation using stable isotope tracers were conducted in the summer mixed-layer community dominated by cyanobacteria, diatoms, and flagellates. Much N_2-fixation occurs at night; moreover, N_2-fixation supported 5-37% of the N-demand for the measured C-fixation by the whole phytoplankton community (< 30 m depth). Rates are values for June, July, and August (mean±SD; three replicates each month).

(a) The average N2-fixation measured from 9 am to 9 pm (open symbols) and from 9 pm to 9 am (closed symbols). The N2-fixation rate during night in the upper 12 m was 53-118% of that in the daytime.
(b) The average C-fixation measured from 9 am to 9 pm (open symbols) and from 9 pm to 9 am (closed symbols).
(c) The depth distribution of the ratio between N_2-fixation rate and N-demand for the whole community. N-demand calculated as the diel C fixation divided by the Redfield ratio (6.6) for each depth. The maximum average value was 19% (range: 8 - 37%) at 12 m depth and the average for the whole euphotic zone was 12% ± 9%.

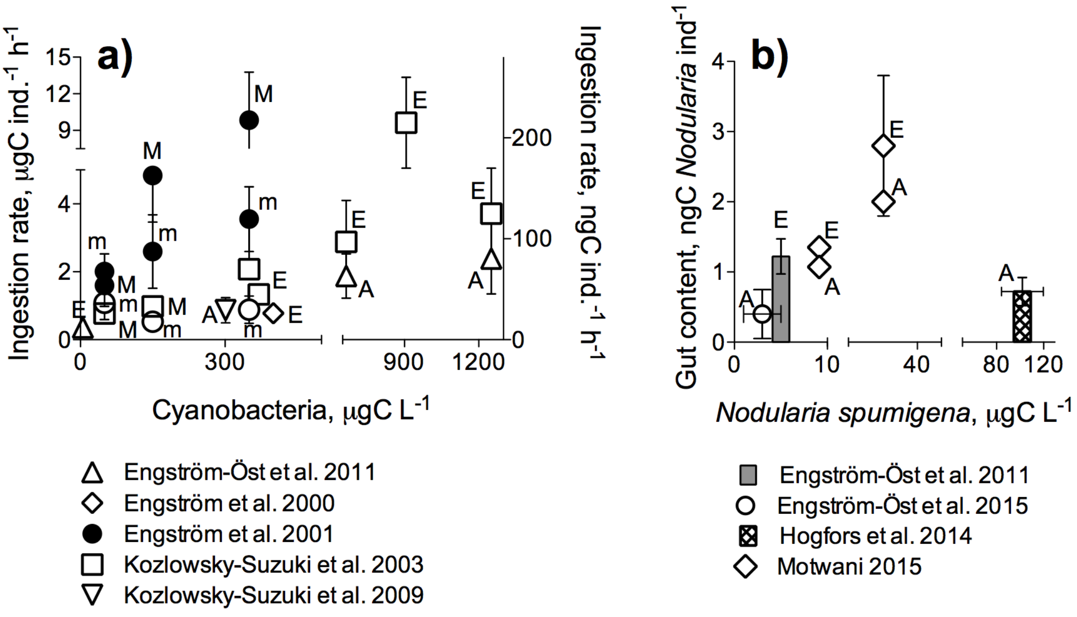

Fig. S2 Grazing by mysids and copepods on *Nodularia spumigena* reported as (a) ingestion rate in feeding experiments and (b) gut content estimated by qPCR in experimental and field studies.

(a) Feeding experiments with *Aphanizomenon* spp. (filled circles) and *Nodularia spumigena* (open symbols) as prey and *Mysis mixta* (circles) and copepods [*Acartia* spp. (A) and *Eurytemora affinis* (E)] as grazers. Two size classes of mysids, juvenile (m) and adult (M), were used. Ingestion rate (mean ± SD) is shown on the left Y-axis for mysids and on the right Y-axis for copepods. Note that most of the experimental studies are conducted at much higher cyanobacteria concentrations than those observed *in situ* (seldom above 20 µgC L^{-1}).

(b) Abundance of *Nodularia spumigena* (mean ± SD) in the stomach bolus of copepods [*Acartia* spp. (A) and *Eurytemora affinis* (E)] measured in the field (open symbols) and in feeding experiments (bars), as a function of the concentration of the cyanobacterium. See Appendix S3 for details on Motwani 2015.

Appendix S1. Monitoring of phyto- and zooplankton in the northern Baltic proper and analysis of long-term trends in zooplankton δ^{15}N in relation to cyanobacteria abundance

Zooplankton samples have been collected within the Swedish National Marine Monitoring Programme (SNMMP) at station B1 (outside Askö Biological Station, northern Baltic Proper; N 58° 48' 18, E 17° 37' 52, bottom depth 38 m), during 1976-2010. The samples were collected by vertical bottom to surface tows, using WP2 net (90 µm mesh size, mouth opening 0.25 m^2) as specified in the monitoring guidelines (HELCOM 2008), preserved in borax-buffered 4% formalin, and stored in the dark, at room temperature. In these samples, mesozooplankton were analysed following the standard protocol of the Baltic Sea Monitoring Programme (HELCOM 1988); biomass was calculated using individual species- and stage-specific weights (Hernroth 1985). Replicate subsamples (Kott 1953) were counted (≥500 specimens) with an inverted microscope (Leitz fluovert FS, Leica) at 80× magnification. Copepods were classified according to species, developmental stage (nauplii, copepodites CI-III, CIV-V, and adults), and sex, whereas cladocerans were classified to species, maturity (females) and sex.

The archival zooplankton samples were used to analyse stable isotopes (SIA) in crustacean zooplankton. For each year, a composite crustacean zooplankton sample for SIA was prepared (25-30 copepods and cladocerans sample^{-1} at approximately 1:1 ratio) using material collected in June-August (5-6 samples per year) and pooled within a year. Thus, each zooplankton sample for SIA represented crustacean zooplankton without an effect of varying proportions of different groups during summer. These zooplankton samples were used for the retrospective SIA to infer temporal changes in zooplankton δ^{15}N isotopic composition.

The samples were dried at 60 ^{o}C for 24 h and analysed using continuous-flow isotope mass spectrometry provided in automated NC analysis (ANCA) SL 20-20, PDZ Europa at the Stable Isotope Facility, UC Davis, U.S.A. The standard reference materials were Vienna PDB and atmospheric N2. Measurement precision determined for standards was ±0.1‰ for carbon and ±0.3‰ for nitrogen isotopes.

Sampling and analysis of phytoplankton were conducted at the same station as a part of SNMMP, following HELCOM guidelines Helcom (2008). Briefly, samples were taken with a hose from the upper 16 m and preserved in acidic Lugol solution. For analysis, the samples were settled in Utermöhl chamber and examined using a NIKON inverted microscope with phase contrast. Phytoplankton (including cyanobacteria) were counted in diagonals or on the half/whole chamber bottom, cell volume was calculated from size measurements. Biovolumes of phytoplankton cells were calculated using Olenina et al. (2006) and the HELCOM taxa-specific biovolume table http://www.ices.dk/marine-data/Documents/ENV/PEG_BVOL.zip.

Appendix S2. Analysis of trophic diversity measured as isotopic niche in the deposit-feeding amphipods in relation to cyanobacteria bloom intensity in the northern Baltic proper.

Samples of the deposit-feeding amphipod *Monoporeia affinis* were collected in the Himmerfjärden Bay, northern Baltic proper (58°59’N and 17°43’E) during five years (2000, 2003, 2006, 2008, 2011) within the program Himmerfjärden Eutrophication Study. Phytoplankton were sampled and analysed bi-weekly throughout the summer as described in Appendix S1, whereas benthic sampling was carried out every third year in October throughout the bay. Six benthic stations close to the phytoplankton monitoring station H3 were selected and 15 amphipods per station, if available (macrofauna abundance in samples varied largely between years and stations), were analysed for stable carbon and nitrogen isotopes (Karlson et al., unpubl.). To quantify trophic level and resource breadth of a population, isotopic niche was used calculated as the convex hull area, which is the total area encompassed by all points on a $\delta^{13}C$–$\delta^{15}N$ bi-plot combining all individual samples (Layman et al. 2007). Larger values of isotopic niche suggest greater trophic diversity and dietary breadth.

Appendix S3. Analysis of direct grazing on cyanobacteria and zooplankton $\delta^{15}N$ in relation to cyanobacteria abundance in the northern Baltic proper

Zooplankton samples have been collected at station BY31 (Landsort Deep, N 58° 35' 00, E 18° 14' 00, bottom depth 454 m) during May-September 2011. The samples were collected by vertical bottom to surface tows, using WP2 net (90 µm mesh size, mouth opening 0.25 m^2) and size fractionated using sieves and separation tower into 100-200 µm (nauplii and rotifers), 200-500 µm (cladocerans and younger copepodites) and >500 µm (older copepodites) size classes. These samples were split and used for SIA and molecular diet analysis.

Two zooplankton sub-samples for each size-fraction were placed into pre-weighed tin-capsules and sample weight (DW, 0.9 ± 0.3; mean ± SD) was measured on a Sartorius balance with a sensitivity of 1 µg prior to SIA. The samples were dried at 60 ^{o}C for 24 h and analysed using continuous-flow isotope mass spectrometry provided in automated NC analysis (ANCA) SL 20-20, PDZ Europa at the Stable Isotope Facility, UC Davis, U.S.A. The standard reference materials were Vienna PDB and atmospheric N_2. Measurement precision determined for standards was ±0.1‰ for carbon and ±0.3‰ for nitrogen isotopes.

To quantify *Nodularia spumigena* in zooplankton guts, a real-time qPCR assay was applied using *Nodularia*-specific primers as described in Engström-Öst et al. (2011). The known number of animals were picked from each size fraction; 7-10 copepods, 10-15 cladocerans and 20-25 nauplii and rotifers per sample were analysed. All samples were analysed in duplicates.

Sampling and analysis of phytoplankton were conducted at the same station as a part of SNMP; see Appendix S1 for details on sampling and sample analysis.

Appendix S4. Analysis of biochemical indices for growth and body condition in the amphipods exposed to cyanobacteria-enriched diet.

Amphipods were exposed to sediment with and without summer bloom (dominated by *Nodularia spumigena*). After a one month incubation, growth indices were measured. As a proxy of growth capacity, we used RNA:DNA ratio, which increases with increasing protein synthesis rate (Lang et al. 1965); it has been successfully applied in various crustaceans, including amphipods (Gorokhova et al. 2010; Ryan et al. 2012). To focus on changes in protein-rich muscle tissue of the experimental animals, nucleic acids were measured using the 6th pereopod dissected from five individuals per experimental container (n=5). The measurements were done using RiboGreen assay according Gorokhova and Kyle (2002). Results from meiofaunal growth using the same experimental set-up is reported in Nascimento et al. (2009).

References:

Degerholm, J., K. Gundersen, B. Bergman, and E. Söderbäck. 2008. Seasonal significance of N-2 fixation in coastal and offshore waters of the northwestern Baltic Sea. *Marine Ecology Progress Series* 360: 73-84

Engström, J., M. Koski, M. Viitasalo, M. Reinikainen, S. Repka, and K. Sivonen. 2000. Feeding interactions of Eurytemora affinis and Acartia bifilosa with toxic and non-toxic Nodularia sp. *Journal of Plankton Research* 22: 1403–1409.

Engström, J., M. Viherluoto, and M., Viitasalo. 2001. Effects of toxic and non-toxic cyanobacteria on grazing, zooplanktivory and survival of the mysid shrimp *Mysis mixta*. *Journal of Experimental Marine Biology and Ecology* 257: 269-280.

Engström-Öst, J., H. Hogfors, R. El-Shehawy, B. De Stasio, A. Vehmaa, and E. Gorokhova. 2011. Toxin producing cyanobacterium *Nodularia spumigena*, potential competitors and grazers: testing mechanisms of reciprocal interactions in mixed plankton communities. *Aquatic Microbial Ecology* 62: 39-48.

Engström-Öst, J., A. Brutemark, A. Vehmaa, N.H. Motwani, and T. Katajisto. 2015. Consequences of a cyanobacteria bloom for copepod reproduction, mortality and sex ratio. *Journal of Plankton Research*, 1-11. doi:10.1093/plankt/fbv004.

Gorokhova, E., and M. Kyle 2002. Analysis of nucleic acids in *Daphnia*: development of methods and ontogenetic variations in RNA-DNA content. *Journal of Plankton Research* 24: 511-522

Gorokhova, E., M. Löf, H. Halldorsson, M. Lindström, T. Elfwing, U. Tjärnlund, and B. Sundelin. 2010. Assessing differential response of *Monoporeia affinis* to hypoxia and contaminants using multiple biomarkers of oxidative stress. *Aquatic Toxicology* 99: 263-274.

Gustafsson, Ö., J. Gelting, P. Andersson, U. Larsson, and P. Roos. 2013. An assessment of upper ocean carbon and nitrogen export fluxes on the boreal continental shelf: A 3-year study in the open Baltic Sea comparing sediment traps, 234Th proxy, nutrient, and oxygen budgets. *Limnology and Oceanography: Methods* 11: 495–510

Helcom 2008 Manual for Marine Monitoring in the COMBINE Programme of HELCOM, Annex 6: Guidelines concerning phytoplankton species composition, abundance and biomass. Available: http://www.helcom.fi/action-areas/monitoring-and-assessment/manuals-and-guidelines/combinemanual, Accessed 2013 Oct 17.

HELCOM 1988. Guidelines for the Baltic monitoring programme for the third stage. Part D. Biological determinands. Baltic Sea Environment Proceedings No 27D. Helsinki Commission, Helsinki.

Hernroth, L. 1985. Recommendations on methods for marine biological studies in the Baltic Sea. Mesozooplankton biomass assessment. *The Baltic Marine Biologists, Publ. 10.*

Hogfors, H. N.H. Motwani, S. Hajdu, R. El-Shehawy, T. Holmborn, A. Vehmaa, J. Engström-Öst, A. Brutemark, et al. 2014. Bloom-forming cyanobacteria support copepod reproduction and development in the Baltic Sea. *PLoS ONE* 9: e112692

Kott, P. 1953. Modified whirling apparatus for the subsampling of plankton. *Australian Journal Marine and Freshwater Research 4: 387–393*

Karlson, A.M.L., E. Gorokhova, and R. Elmgren. 2014. Nitrogen fixed by cyanobacteria is utilized by deposit-feeders. *PLoS ONE* 9: e104460.

Kozlowsky-Suzuki, B., M. Karjalainen, M. Lehtiniemi, J. Engström-Öst, M. Koski, and P. Carlsson. 2003. Feeding, reproduction and toxin accumulation by the copepods *Acartia bifilosa* and *Eurytemora affinis* in the presence of the toxic cyanobacterium *Nodularia spumigena. Marine Ecology Progress Series* 249: 237-249.

Kozlowsky-Suzuki, B., M. Koski, E., Hallberg, R. Wallén, and P. Carlsson. 2009. Glutathione transferase activity and oocyte development in copepods exposed to toxic phytoplankton. *Harmful Algae* 8: 395-406.

Lang, C.A., H.Y. Lau, and D.J. Jefferson. 1965. Protein and nucleic acid changes during growth and aging in the mosquito. *Biochemical Journal* 95: 372.

Larsson, U., S. Hajdu. J. Walve, and R. Elmgren. 2001. Estimating Baltic nitrogen fixation from the summer increase in upper mixed layer total nitrogen. *Limnology and Oceanography 46: 811-820.*

Layman, C.A., D.A. Arrington, C. G. Montana, and D.M. Post. 2007. Can stable isotope ratios provide for community-wide measures of trophic structure? *Ecology* 88: 42–48.

Motwani, N.H. 2015. Trophic complexity of zooplankton-cyanobacteria interactions in the Baltic Sea: insights from molecular diet analysis. PhD thesis. Stockholm, Sweden: Stockholm University.

Nascimento, F.J.A., A.M.L. Karlson, J. Näslund, and E. Gorokhova. 2009. Settling cyanobacterial blooms do not improve growth conditions for soft bottom meiofauna. *Journal of Experimental Marine Biology and Ecology* 368: 138-146.

Ohlendieck, U., A. Stuhr, and H, Siegmund. 2000. Nitrogen fixation by diazotrophic cyanobacteria in the Baltic Sea and transfer of the newly fixed nitrogen to picoplankton organisms. *Journal of Marine Systems* 25: 213-219.

Ohlendieck, U., K. Gundersen, M. Meyerhöfer, P. Fritsche, K. Nachtigall, and B. Bergmann. 2007. The significance of nitrogen fixation to new production during early summer in the Baltic Sea. *Biogeosciences* 4: 63-73

Olenina, I., S. Hajdu, L. Edler, A. Andersson, N. Wasmund, S. Busch, J. Göbel, S. Gromisz, S. Huseby, M. Huttunen, A. Jaanus, P. Kokkonen, I. Ledaine, and E. Niemkiewicz. 2006. Biovolumes and size-classes of phytoplankton in the Baltic Sea. *HELCOM Baltic Sea Environment Proceedings 106, Helsinki*

Rahm, L., A. Jonsson, and F. Wulf. 2000. Nitrogen fixation in the Baltic proper: an empirical study. *Journal of Marine Systems* 25: 239-248

Rolff, C., L. Almesjö, and R. Elmgren. 2007. Nitrogen fixation and the abundance of the diazotrophic cyanobacterium Aphanizomenon sp. in the Baltic Proper. *Marine Ecology Progress Series 332: 107-118.*

Ryan, D.J., M.S. Sepúlveda, T.F. Nalepa, and T.O. Höök. 2012. Spatial variation in RNA:DNA ratios of *Diporeia* spp. in the Great Lakes region. *Journal of Great Lakes Research* 38: 187–195

Stal, L.J., and A.E. Walsby. 1998. The daily integral of nitrogen fixation by planktonic cyanobacteria in the Baltic Sea. *New Phytologist 139: 665-671*

Stal, L.J., P. Albertano, B. Bergman, K. Brockeld, J.R. Gallone, P.K. Hayes, K. Sivonen, and A.E. Walsby, 2003. BASIC: Baltic Sea cyanobacteria. An investigation of the structure and dynamics of water blooms of cyanobacteria in the Baltic Sea- responses to a changing environment. *Continental Shelf Research* 23: 1695–1714.

Wasmund, N., M. Voss, and K. Lochte. 2001. Evidence of nitrogen fixation by non-heterocystous cyanobacteria in the Baltic Sea and re-calculation of a budget of nitrogen fixation. *Marine Ecology Progress Series 214: 1-14*

Wasmund, N., G. Nausch, B. Schneider, K. Nagel, and M. Voss. 2005. Comparison of nitrogen fixation rates determined with different methods: a study in the Baltic Proper. *Marine Ecology Progress Series 297: 23-31.*
